# Obsessive-Compulsive (Anankastic) Personality Disorder in the ICD-11: A Scoping Review

**DOI:** 10.3389/fpsyt.2021.646030

**Published:** 2021-03-16

**Authors:** Julija Gecaite-Stonciene, Christine Lochner, Clara Marincowitz, Naomi A. Fineberg, Dan J. Stein

**Affiliations:** ^1^Laboratory of Behavioral Medicine, Neuroscience Institute, Lithuanian University of Health Sciences, Palanga, Lithuania; ^2^South African Medical Research Council Unit on Risk and Resilience in Mental Disorders, Department of Psychiatry, Stellenbosch University, Stellenbosch, South Africa; ^3^National Obsessive Compulsive Disorders Specialist Service, Hertfordshire Partnership University National Health Service Foundation Trust, University of Hertfordshire, Hatfield, United Kingdom; ^4^South African Medical Research Council Unit on Risk and Resilience in Mental Disorders, Department of Psychiatry and Neuroscience Institute, University of Cape Town, Cape Town, South Africa

**Keywords:** ICD-11, personality disorders, obsessive-compulsive personality, anankastic personality, anankastia, DSM-5, diagnosis and classification, domains

## Abstract

**Introduction:** With the shift from a categorical to a dimensional model, ICD-11 has made substantial changes to the diagnosis of personality disorders (PDs), including obsessive-compulsive (anankastic) personality disorder (OCPD). The ICD-11 PD model proposes a single diagnosis of PD with specifications regarding severity and domains. However, a systematic overview of ICD-11 anankastia is lacking. In this review we address the reformulation of the OCPD diagnosis in the ICD-11, and draw comparisons with the DSM-5, with a particular focus on diagnostic validity and clinical utility. We hypothesized that the ICD-11 PD model provides a diagnostically valid and clinically useful approach to OCPD, with specific emphasis on the anankastia domain as the primary trait qualifier.

**Methods:** Literature published from 2010 to 2020 was systematically searched using the PubMed/MEDLINE, PsychInfo, Cochrane, and Web of Sciences search engines, in order to find all articles that addressed ICD-11 anankastia. Relevant articles were collated, and themes of these articles subsequently extracted.

**Results:** Out of the 264 publications identified, 19 articles were included in this review. Four themes were identified, namely (a) overlap of DSM-5 OCPD with the ICD-11 PD model, (b) the factorial structure of the ICD-11 PD model with respect to the anankastia domain, (c) the clinical utility of the ICD-11 PD model, and (d) comparison of the ICD-11 PD model of anankastia with the DSM-5 alternative model for OCPD.

**Conclusions:** The ICD-11 anankastia domain overlaps with DSM-5 OCPD traits, and the factor analyses of the ICD-11 PD model further support the diagnostic validity of this domain. There is some support for the clinical utility of the ICD-11 PD model of anankastia but further studies are needed, including of its relationship to obsessive-compulsive and related disorders.

## Introduction

Obsessive-compulsive personality disorder (OCPD) in the *Diagnostic and Statistical Manual of Mental Disorders* (5th edition, DSM-5) (1) or anankastic personality disorder in the *International Classification of Diseases* (10th edition, ICD-10) (2), is characterized by an excessive preoccupation with orderliness, mental and interpersonal control, and perfectionism at the expense of efficiency, openness and flexibility. As with other personality disorders (PDs), this maladaptive pattern has an onset in adolescence or early adulthood, is stable over time, and markedly affects functioning resulting in significant distress and impairment (1). Even though obsessive-compulsive personality traits affect around 2–7% of the healthy population (3–6) and 23–26% of clinical populations (7, 8), OCPD is still a relatively under-diagnosed and under-researched disorder (9, 10).

The operationalization of PDs, including OCPD, in both the DSM and the ICD taxonomies, has been a subject of debate (9, 11, 12). In particular, the categorical model for PD diagnosis has been criticized, with some arguing that this approach lacks diagnostic validity and has limited clinical utility (13). Criticism regarding diagnostic validity emphasizes that personality traits are dimensional (rather than categorical), the high comorbidity of PDs in general, and the heterogeneity of OCPD in particular. The heterogeneity of OCPD is emphasized by data which fail to find specific hallmark factors underlying DSM-5 OCPD. Criticism regarding clinical utility emphasizes that inclusion of PDs in DSM-III and the ICD-10 has not diminished the substantial underdiagnosis of these conditions (9, 13–19).

A proposal to move to a dimensional conceptualization of PD, including OCPD, was put forward by the DSM-5 Personality and Personality Disorders Work Group (20), which outlined an Alternative Model of PD (AMPD). However, the final DSM-5 decision was to retain the categorical model of PDs, and the AMPD was confined to Section III of the DSM-5 for further research (1). While the DSM-5 AMPD does not include a domain for obsessive-compulsive personality traits, it retains six categories of PDs, one of which is OCPD (17, 21, 22). The compulsivity domain was not included in the final model, as this was considered to be an diametrically opposite trait to the disinhibition domain (20).

In contrast, ICD-11 has moved away from a categorical framework of PDs to an entirely dimensional system (23) without categorical PD diagnoses. According to the ICD-11 guidelines, the clinician first determines whether the individual has a PD (24). Thereafter the level of severity is assessed, and labeled as mild, moderate or severe (24). In the final step, the maladaptive personality is described in terms of the trait qualifiers including anankastia (24), which is characterized as “a narrow focus on one's rigid standard of perfection and of right and wrong” as well as controlling behavior regarding oneself, others and situations in order to “ensure conformity to these standards” (25). The ICD-11 PD model and the DSM-5 AMPD have a great deal in common, including agreeing on four out of five trait domains (i.e., negative affect, detachment, dissociality/antagonism, and disinhibition but not anankastia).

A number of publications have addressed the ICD-11 conceptualization of PDs in general, and a number of studies have focused on the ICD-11 domain of anankastia in particular. However, we are not aware of any review that has synthesized the literature on ICD-11 anankastia. Given the recency of this conceptualization, we chose to conduct a scoping review to assess the existing body of literature and identify knowledge gaps (26).

The current scoping review aims to provide a comprehensive overview and synthesis of empirical research on ICD-11 anankastia to date, with a particular focus on diagnostic validity and clinical utility. Due to the limited number of studies, the final pool of selected literature was not subjected to restrictions in terms of study population, intervention type, comparators or outcomes of interest (PICO). Our hypothesis was that the ICD-11 PD model is a diagnostically valid and clinically useful approach to OCPD.

## Methods

**Figure 1:**
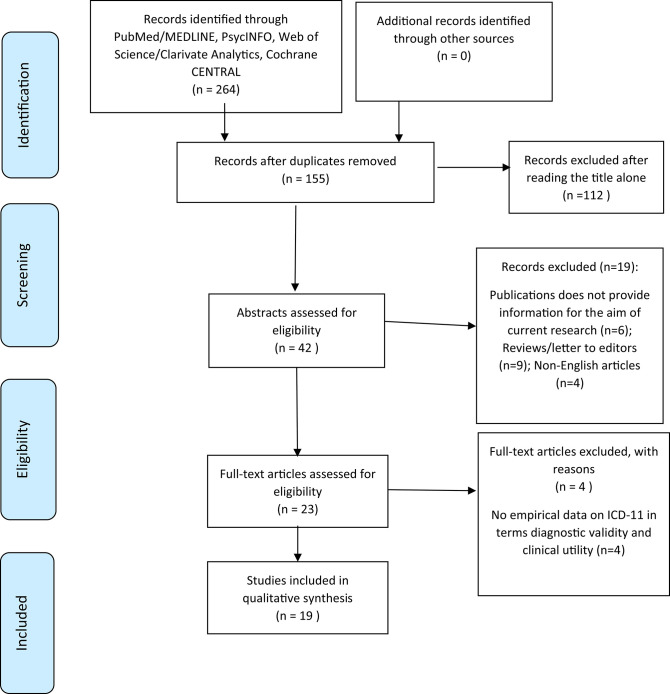
PRISMA 2009 flow chart of study selection. Query: (ICD-11 AND personality disorder^*^ AND (“obsessive compulsive personality” OR anankastia OR “Anankastic personality”)) OR (ICD-11 AND “personality disorder^*^” AND trait qualifier^*^) OR (ICD-11 AND “personality disorder^*^” AND domain^*^). Copyright: Moher et al. (28).

A systematic search was conducted using PubMed/MEDLINE, PsycInfo, Cochrane, and Web of Sciences electronic databases in order to identify relevant peer-reviewed manuscripts published from January 2010 to October 2020. The search was undertaken in accordance with the Preferred Reporting Items for Systematic reviews and Meta-Analysis (PRISMA) (27). We used the following search strings: (1) ICD-11 AND personality disorder^*^ AND (“obsessive compulsive personality” OR anankastia OR “anankastic personality”); (2) ICD-11 AND “personality disorder^*^” AND trait qualifier^*^; (3) ICD-11 AND “personality disorder^*^” AND domain^*^ (Figure 1).

Studies were considered for inclusion if they addressed the classification of OCPD within the ICD-11 PD model. This included studies of the ICD-11 anankastia domain. There were no restrictions to inclusion criteria regarding country of origin, sample size, or PICO due to the relative scarcity of publications relevant to the study hypothesis. Due to the nature of the hypothesis, selection of studies was limited to those with a solely empirical research design (i.e., descriptive, correlational or experimental studies). Publications that were not available in English (29–32) were excluded. Relevant articles were collated, and themes of these articles were extracted. A methodological quality check was completed on the publications that were included in the final pool based on methodology checklists of NICE Clinical Guidelines (33, 34).

## Results

The search yielded 19 studies for review (Table 1), all of which had adequate methodological quality, as defined by NICE checklist (33). Based on these studies, four themes were identified, namely (a) overlap of DSM-5 OCPD with the ICD-11 PD model, (b) the factorial structure of the ICD-11 PD model with respect to the anankastia domain, (c) the clinical utility of the ICD-11 PD model, and (d) comparison of the ICD-11 PD model of anankastia with the DSM-5 alternative model for OCPD.

**Table 1:** Characteristics of selected studies.

**References**	**Country**	**Study design**	**Study population (sample size, *n*)**	**Proportion of women [*n* (%)]**	**Mean age ± standard deviation**	**PD assessment**	**Main results regarding the ICD-11 classification (OCPD or anankastia domain)**
Kim et al. (35)	South Korea	Field trial	Patients with PD (*n* = 124)	74 (59.68%)	30.7 ± 11.82	PAS SAPAS-SR NEO-FFI	The patients with OCPD were mainly distributed in the anankastic-obsessional domain; Highest predictive accuracy found in the anankastic domain; Anankastic domain showed good discriminant validity Convergent-divergent validities were not supported for the anankastic domain
Mulder et al. (22)	New Zealand	Cross-sectional (from five randomized control trials)	Psychiatric patients diagnosed with major depression (*n* = 606)	378 (62.4%)	34.2 ± 11.1	SCID II ICD-11 PD domains assessed by two individual clinicians	Anankastic domain mainly consists of all eight criteria for the OCPD (DSM-5). Criterion 7 (miserliness) is relatively weakly related to this domain; One criterion of Avoidant PD (reluctance to take risks) fell under anankastia domain.
Bach et al. (36)	Denmark	Cross-sectional	Derivation sample (*n* = 1,541): Psychiatric out-patients (*n* = 615) Community sample (*n* = 925) Replication sample –undergraduate students (*n* = 637)	1,248 (80.9 %) 357 (56%)	32.64 ± 12.04 19.36 ± 1.64	PID-5	Acceptable discriminant validity for anankastia domain in replication sample; Domain of anankastia emerged from negative affectivity (facet perseveration) and disinhibition (facets rigid perfectionism and distractibility) domains of the AMPD DSM-5;
Lotfi et al. (37)	Iran	Cross-sectional	Community sample (*n* = 285)	188 (66%)	30 ± 8.29	PID-5	Anankastia domain emerged from negative affectivity (facets perseveration, hostility) and disinhibition (facet rigid perfectionism) domains of the AMPD DSM-5.
Bach et al. (17)	Denmark	Cross-sectional	Psychiatric outpatients (*n* = 226)	131 (58%)	32.54 ± 10.02	SCID-II PID-5	ICD-11 model was superior to the DSM-5 AMPD in capturing OCPD; Anankastia domain was specified using facets of the DSM-5 AMPD rigid perfectionism (domain *low* Disinhibition) and perseveration (domain Negative affectivity) The ICD-11 domain of anankastia showed the strongest prediction of OCPD; OCPD was also predicted by domains of negative affectivity and low disinhibition.
Oltmanns et al. (38)	US	Cross-sectional	Clinical sample: Participants with previous or current mental health problems Study I: *n* = 259 Study II: *n* =285	176 (68%) 188 (66%)	35.7 ± 11.0 35.1 ± 10.9	PiCD EPQ-R 5-DPT CAT-PD-SF SCID-II	Satisfactory discriminant validity of anankastia domain was found; Anankastic domain converged negatively with disinhibition domain at a medium effect size.
Pesic et al. (39)	Serbia	Cross-sectional	Psychiatric patients with diagnosed PD (*n* = 223)	149 (67%)	37.6 ± 13	Five ICD-11 PD domains retrieved from computed factor analysis	-Anankastic domain mainly consists of all eight criteria for OCPD (DSM-5)
McCabe et al. (40)	US	Cross-sectional	Community sample (*n* = 300)	162 (54%)	36.51 ± 10.36	SASPD PiCD PID-5 LPFS-BF BPS MAPP WISPI	Excellent convergent validity for anankastia domain Anankastia domain was associated with facets of rigid perfectionism (domain disinhibition) and perseveration (domain negative affectivity) from DSM-5 AMPD; Bipolar anankastia and disinhibition factor remained, suggesting anankastia and disinhibition are opposite to one another
Oltmanns et al. (41)	US	Cross-sectional	Clinical sample: Participants with previous or current mental health problems Study I: *n* = 311 Study II: *n* = 148 Study III: *n* = 301	205 (66%) 92 (62%) 184 (61%)	36.6 ± 12.0 35.6 ± 12.5 36.5 ± 10.7	FFiCD PiCD PID-5 FFMPD Pool-NEO-120 FFF FFMRF	Recommendation of four factors, where anankastia and disinhibition formed a single bipolar factor. The FFiCD facets of perfectionism, inflexibility, and workaholism loaded with the ICD-11 anankastia.
Carnovale et al. (42)	Canada	Cross-sectional	Student sample (*n* = 518)	366 (70.66%)	19.26 ± 3.05	PiCD MMPI-2-RF	Suggestion of 4-factor solution, with the one factor representing a bipolar continuum of Anankastia and Disinhibition. The largest absolute correlation was between disinhibition and anankastia scores, the smallest was between dissocial and anankastia scores; Anankastia domain showed low discriminant validity
Bach et al. (43)	Denmark	Cross-sectional	Psychiatric patients (*n* = 238)	174 (73%)	33.21 ± 15.48	PiCD-IRF	Two possible factorial solutions: 4-factor model included bipolar domain of anankastia/disinhibiton, while 5-factor solution included two separate unipolar domains of anankastia and disinhibition; Discriminant validity of anankastia domain satisfactory.
Sellbom et al. (21)	Canada	Cross-sectional	Psychiatric outpatients (*n* = 343)	172 (50.2%)	38.94 ± 10.17	PID-5 SCID-II-PQ MMPI-2-RF NEO PI-R	Anankastia was most strongly correlated with OCPD and was the best predictor of OCPD Anankastia domain was linked with facets of rigid perfectionism (domain disinhibition) and perseveration (domain negative affectivity) from AMPD DSM-5;
Gutierrez et al. (44)	Spain	Cross-sectional	Community sample (*n* = 2,522) Clinical sample (n = 797)	2,522 (59.2%) 558 (70.7%)	39.8 ± 19.0 41.7 ± 13.6	PiCD SASPD	Found 4 factor-solution, where anankastia and disinhibition formed a single bipolar factor.
Somma et al. (45)	Italy	Cross-sectional	Community sample (*N* = 1,122)	867 (77.3%)	31.94 ± 12.44	PiCD FFMPI BFI PID-5-SF MDPF	Found 4 factor-solution, where anankastia and disinhibition formed a single bipolar factor.
Kim et al. (46)	South Korea	Cross-sectional	Female students (*n* = 334) Psychiatric out-patients (*n* = 75) *A subset of the sample (n = 210):* Psychiatric patients (*n* = 75) Female students (*n* = 135)	337 (100 %) 49 (65.33%) 135 (100 %) 49 (65.33%)	23.7 ± 7.3 25.8 ± 9.5	PAQ-11 PBQ-SF NEO-FFI SAPAS-SR PID-5 SF	Found 5 factor-solution, where anankastia and disinhibition formed separate unipolar domains; Anankastia domain was correlated with obsessive–compulsive personality belief
Tarescavage et al. (47)	US	Cross-sectional	Student sample (*n* = 328)	178 (54.27%)	19.3 ± 1.4	PiCD MMPI-2-RF CAT-PD-SF	Found 4 factor-solution, where anankastia and disinhibition formed a single bipolar factor.
Aluja et al. (48)	Spain	Cross-sectional	Community sample (*n* = 1,229)	651 (52.97%)	39.63 ± 17.81	ZKA-PQ/SF PID-5-SF PiCD	Found 4 factor-solution, where anankastia and disinhibition formed a single bipolar factor.
Bach and Abiddine (49)	Algeria	Cross-sectional	Student sample (*n* = 638)	433 (67.9%)	21.3 ± 3.05	PID-5-BF	Revealed four-factor structure that aligned with the ICD-11 trait domain qualifiers, including a single factor dedicated to Disinhibition vs. low Anankastia
^*^Hansen et al. (50)	Denmark	Cross-sectional	Psychiatric patients (*n* = 163)	144 (69.9%)	33.15 ± 14.88	PD administered by 163 clinicians based on the given ICD-10 and ICD-11 guidelines ^*^additional questionnaire CUQ	The ICD-11 dimensional PD model was rated as slightly more useful than former ICD-10 framework No specific information regarding the ICD-11 Anankastia domain and its relationship to OCPD is given

*OCPD, Obsessive compulsive personality disorder; ICD-11, The International Classification of Diseases 11th revision; PD, personality disorder; AMPD DSM-5, Alternative Model for PD from Diagnostic and Statistical Manual of Mental Disorders Fifth Edition; PAS, Personality Assessment Schedule; SAPAS-SR, Standardized Assessment of Personality-Abbreviated Scale, self-report form; NEO-FFI, NEO Five-Factor Inventory; SCID-II, Structured Clinical Interview for DSM-IV; PID-5, Personality Inventory for DSM-5; PiCD, Personality Inventory for the ICD-11; EPQ-R, The Eysenck Personality Questionnaire-Revised; 5-DPT, 5-Dimensional Personality Test; CAT-PD-SF, CAT-Personality Disorder Scales Static Form; SASPD, Standardized Assessment of Severity of Personality Disorder; LPFS-BF, Levels of Personality Functioning Scale-Brief Form; BPS, Borderline Pattern Specifier; MAPP, Multi-Source Assessment of Personality Pathology; WISPI, The Wisconsin Personality Disorder Inventory; FFiCD, Five-Factor Personality Inventory for the ICD-11; FFMPD, five-factor model of personality disorder scales; Pool-NEO-120, International Personality Item; FFF, The Five Factor Form; FFMRF, Five Factor Model Rating Form; MMPI-2-RF, Minnesota Multiphasic Personality Inventory−2–Restructured Form; PiCD-IRF, Informant-report form of the Personality Inventory for the ICD-11; SCID-II-PQ, Structured Clinical Interview for DSM–IV Axis II Disorders—Personality Questionnaire; MMPI-2-RF, Minnesota Multiphasic Personality Inventory−2 Restructured Form; NEO PI-R, Revised NEO Personality Inventory; FFMPI, Five-Factor Model Personality Index; BFI, Big Five Inventory; PID-5-SF, Personality Inventory for DSM−5. Short Form; PID-5-BF, Personality Inventory for DSM−5. Brief Form; MDPF, Measure of Disordered Personality Functioning, PAQ-11, Personality Assessment Questionnaire for the ICD-11; PBQ-SF, Short Form The Personality Belief Questionnaire—Short Form; ZKA-PQ/SF, The Short Form of the Zuckerman–Kuhlman–Aluja Personality Questionnaire; CUQ, Clinical utility questionnaire*.

* *Additional study on clinical utility regarding the ICD-11 PD framework*.

Most of the studies (*n* =11) were published in 2020. Eight (8) studies were conducted in Europe, six in North America, three in Asia, one in Africa and one in New Zealand. Overall, nine studies were conducted on a clinical psychiatry sample, eight were conducted in the general population, and two studies were undertaken in both of these groups. Sample size ranged from 124 in a study of patients with PD (35) to 2,522 in a study of participants in a community sample (44). Most of the studies (*n* = 18) addressed the question of diagnostic validity, while a single study examined the clinical utility of the ICD-11 PD model.

### Overlap of DSM-5 OCPD With the ICD-11 PD Model

Five (5) empirical studies investigated the overlap of DSM-5 OCPD with ICD-11 PD domains (17, 21, 22, 35, 39). The largest of these studies examined the factorial structure of the ICD-11 PD model in 606 patients with major depression (22). The authors reported that all of the DSM-5 OCPD criteria (i.e., maladaptive preoccupation with details, perfectionism, excessive devotion to work, over-conscientiousness, inability to discard things, reluctance to delegate the tasks, miserliness, and rigidity) fell in the ICD-11 domain of anankastia. An additional symptom of avoidance of, or reluctance to take risks (found in the DSM-5 avoidant PD), also fell in the ICD-11 anankastia domain (22).

In an earlier study (35) conducted in 124 patients with PD defined by ICD-11 terms, a linear discriminant analysis revealed that DSM-5 OCPD traits were mainly distributed in the ICD-11 anankastia domain. In addition, the ICD-11 anankastia domain showed the highest predictive accuracy of all the ICD-11 PD domains, as well as good discriminant validity, but had weak convergent-divergent validity. In particular, the ICD-11 trait qualifiers correctly classified 100% of anankastic cases within the originally grouped individuals (35). However, the anankastic trait qualifier was not significantly linked with any of the traits of the five-factor model (51) as expected (i.e., neuroticism, extraversion, openness, agreeableness and conscientiousness) (35). Similar findings emerged in the later studies by Bach et al. (17), Pesic et al. (39) and Sellbom et al. (21). Specifically, when examining the multidimensional structure of the ICD-11 PD model in 343 psychiatric outpatients (39), all DSM-5 OCPD criteria fell in the ICD-11 anankastia domain. In the two other studies with psychiatric patients (*n* = 226 and *n* = 223, respectively) that examined associations between ICD-11 anankastia and DSM-5 OCPD, the ICD-11 anankastia domain was more predictive of the presence of the DSM-5 OCPD than of other PDs (17, 21). In addition, there is some evidence that the ICD-11 domains of *low* disinhibition and *high* negative affectivity (17, 21) are additional trait qualifiers that predict OCPD.

### Factorial Structure of the ICD-11 PD Model Regarding Anankastia Domain

Eleven (11) publications reported on the factorial structure of the ICD-11 PD model, indicating a 4-factor solution (41, 42, 44, 45, 47–49), a 5-factor solution (46) or both (38, 40, 43). All of the studies were conducted in either psychiatry samples, general population samples, or both, while the sample size ranged from 162 to 2,522 participants. The ICD-11 PD domains of negative affectivity, dissociality and detachment formed separate factors in all of the studies. In the 4-factor solutions, the anankastia domain and the disinhibition domain fell at two ends of a single factor, with low disinhibition at the one end and high anankastia at the other. Additionally, in a study of 366 students (42), the anankastia domain showed low discriminant validity, while in a study of 174 psychiatric patients (43), the anankastia domain had satisfactory discriminant validity.

### The Clinical Utility of the ICD-11 PD Model

A single study (50) in Denmark reported on the clinical utility of the ICD-11 PD model. PD was evaluated by mental health professionals based on the given ICD-10 and ICD-11 guidelines. In a sample of 163 psychiatric patients with mostly mood and anxiety disorders, psychotic disorders and PD disorders, the ICD-11 PD model was found to be slightly more useful than the ICD-10 in determining the presence of PD. Different professionals had somewhat different views, with psychologists reporting that the ICD-11 PD model was more useful in formulating an effective treatment plan whereas medical doctors and nurses found them equal. Regarding utility for communication with other mental health specialists and description of global personality, there was no difference between the ICD-11 PD model and ICD-10 categorical model. Age and work experience of the clinicians did not influence views regarding the rating of the ICD-10 vs. the ICD-11 clinical application.

### The ICD-11 PD Model vs. the DSM-5 Alternative Model for OCPD

After the introduction of the new ICD-11 PD model, there have been five studies comparing the ICD-11 PD model and the DSM-5 trait based AMPD (17, 21, 36, 37, 40). All of these studies, whether conducted in a psychiatric sample or in a community sample, found a significant correlation between the ICD-11 anankastia domain and the DSM-5 domains of negative affectivity (specifying facet - perseveration) and *low* disinhibition (specifying facet - rigid perfectionism). In a sample of 1,541 individuals comprised of the general population and psychiatric outpatients, the additional trait of distractibility (found in the *low* disinhibition domain) was also associated with the ICD-11 anankastia domain (36). In 285 individuals from a community sample, the trait of hostility (negative affectivity domain) loaded on the ICD-11 anankastia domain (37). In 1,541 psychiatric and healthy participants (36), acceptable discriminant validity was found between the ICD-11 anankastia domain and DSM-5 OCPD. Similarly, in a general population sample of 300 individuals (40), excellent convergent validity between the ICD-11 anankastia domain and the DSM-5 OCPD was documented. In addition, in two studies comprised of 226 (17) and 343 (21) psychiatric outpatients, the ICD-11 domain of anankastia showed the strongest prediction of OCPD.

## Discussion

This scoping review found 19 empirical studies on the ICD-11 anankastia domain. Four themes were identified based on the literature, namely (a) overlap of DSM-5 OCPD with the ICD-11 PD model, (b) the factorial structure of the ICD-11 anankastia domain, (c) the clinical utility of the ICD-11 anankastia domain, and (d) comparison of the ICD-11 PD model of anankastia with the DSM-5 alternative model for OCPD.

As hypothesized, work on the overlap of DSM-5 OCPD with ICD-11 PD model found that the anankastia domain is strongly associated with OCPD traits in both clinical and community samples (21, 22, 25, 35, 39). One study found that the additional symptom of *avoidance of, or reluctance to take risks* (from the DSM-5 avoidant PD) was also associated with the anankastia domain (22). DSM-5 OCPD traits were also associated with the ICD-11 domains of *low* disinhibition and negative affectivity (17, 21). The finding that OCPD traits overlap with different domains is consistent with work demonstrating that OCPD is comorbid with a number of other PDs including avoidant (52), paranoid (52, 53), schizotypal (54), borderline and narcissistic (52) PDs. A dimensional structure for describing maladaptive personality traits may be helpful in addressing the artifactual comorbidity that occurs in a categorical system (13, 55).

Studies on the ICD-11 PD model and its factorial structure suggested using either a five-factor solution or four-factor solutions, resulting in a single *low* disinhibition/*high* anankastia domain (38, 40–49). In this multidimensional structure, OCPD could be distinguished by a high score on anankastia traits, automatically resulting in low disinhibition traits. These findings complement work indicating that individuals with OCPD not only have high anankastia traits but also low disinhibition traits when these two domains are investigated separately (17, 21). In addition, it is also relevant to note inconsistencies regarding the convergent validity of the ICD-11 anankastia domain. Specifically, in a study including 124 patients with PD, convergent validity was not supported (35), while excellent convergent validity was documented within a sample of 300 community members (40). This inconsistency might be a consequence of population and/or methodological differences between studies.

Clinical utility of the ICD-11 PD model for psychiatric patients was observed in a study conducted in Denmark, which provided some evidence that the ICD-11 PD model is slightly more useful than the ICD-10 classification in determining the presence of PD (38). However, the OCPD and anankastia domain were not specifically addressed in this work. In the past mixed views regarding the clinical utility of the ICD-11 PD model have been expressed (36, 56, 57). On the one hand, the ICD-11 PD model was expected to be simpler to use (56) and more feasible for practitioners (36). On the other hand, it might not be easily accepted by practitioners, as thinking dimensionally might be more incommodious and time-consuming for clinicians compared to thinking in categorical terms (57). In addition, regarding clinical utility, several other questions raised in previous studies regarding the ICD-11 PD model were not addressed by the literature, and remain to be answered. First, the issue of arbitrary diagnostic thresholds that has been discussed for categorical diagnostic models (56, 58) may remain, since there is still no clear-cut way of distinguishing abnormal personality traits including anankastic traits in ICD-11 (59). Second, with the introduction of the ICD-11 PD model, it was hoped that PD would be detected more frequently, so addressing the underdiagnosis issue with previous versions of the ICD (60, 61). In the selected studies however, we could not find evidence comparing the ICD-10/DSM-5 with the ICD-11 in terms of detected prevalence of abnormal obsessive-compulsive personality traits. To answer these questions regarding the clinical utility of the ICD-11 PD model, more empirical studies in different regions and samples are needed.

In terms of comparison of the ICD-11 PD model and the DSM-5 AMPD, several relevant studies were found. The DSM-5 AMPD domains of negative affectivity (facets of perseveration, hostility) and *low* disinhibition (rigid perfectionism, distractibility) were found to be predictors of the ICD-11 anankastia domain (17, 21, 36, 37, 40), consistent with the conceptual similarity of the domains of negative affectivity and disinhibition in these nosologies (62). These findings were also in line with views that ICD-11 anankastia, or obsessive-compulsive traits in DSM-5, are the inverse trait of disinhibition, so leading to the omission of such traits in the final DSM-5 AMPD model (20). Nevertheless, perhaps because the ICD-11 PD model contains a separate anankastia domain, the ICD-11 was found to be superior in determining the presence of obsessive-compulsive personality traits in comparison to the DSM-5 AMPD framework (17).

Limitations of this scoping review deserve acknowledgment. In particular, the review was limited to articles written in English, so excluding a number of potentially relevant studies. In addition, key limitations of the literature itself deserve emphasis. First, the methods and instruments to assess the ICD-11 PD domains varied significantly, making it challenging to compare results across studies. Second, studies of the ICD-11 model are limited to only a small number of countries. Third, we found no longitudinal studies of the diagnostic reliability of the ICD-11 over time. Fourth, we found no papers exploring ICD-11 PD and the anankastia domain in individuals with obsessive-compulsive and related disorders. Thus, further longitudinal studies in more diverse cultural cohorts and in both community and clinical populations, using consensus instruments, are warranted.

## Conclusion

OCPD is a common mental health problem that is still relatively under-recognized and lacks empirical investigations. This scoping review suggests that the ICD-11 PD model is a diagnostically valid and clinically useful approach to OCPD. Specifically, the ICD-11 anankastia domain overlaps with DSM-5 OCPD traits, with factor analyses of the ICD-11 PD model further supporting the diagnostic validity of this domain. There is some support for the clinical utility of the ICD-11 PD model with regards to anankastia. Future studies investigating the clinical utility of ICD-11 PD in more diverse clinical and cultural samples are warranted. Finally, further work exploring the overlap of the ICD-11 anankastia domain with DSM-5 obsessive compulsive and related disorders (9) is needed.

## Data Availability Statement

The original contributions presented in the study are included in the article further inquiries can be directed to the corresponding author.

## Author Contributions

DS conceptualized the idea, supervised during the writing process, and provided critical revisions. JG-S conducted the systematic search, prepared the first draft of the manuscript, and revised the manuscript. CL, NF, and CM provided critical revision to its further development. All authors contributed to the article and approved the submitted version.

## Conflict of Interest

JG-S serves as a consultant at FACITtrans. NF declares that in the past 3 years she has held research or networking grants from the ECNP, UK NIHR, EU H2020, MRC, University of Hertfordshire; she has accepted travel and/or hospitality expenses from the BAP, ECNP, RCPsych, CINP, International Forum of Mood and Anxiety Disorders, World Psychiatric Association, Indian Association for Biological Psychiatry, Sun; she has received payment from Taylor and Francis and Elsevier for editorial duties. In the past 3 years, she has accepted a paid speaking engagement in a webinar sponsored by Abbott. Previously, she has accepted paid speaking engagements in various industry supported symposia and has recruited patients for various industry-sponsored studies in the field of OCD treatment. She leads an NHS treatment service for OCD. She holds Board membership for various registered charities linked to OCD. She gives expert advice on psychopharmacology to the UK MHRA. DS has received research grants and/or consultancy honoraria from Johnson & Johnson, Lundbeck, Servier, and Takeda. The handling editor declared a past collaboration with one of the authors DS. The remaining authors declare that the research was conducted in the absence of any commercial or financial relationships that could be construed as a potential conflict of interest.
